# 1521. High frequency of vitamin D deficiency in hospitalized patients with HIV: A prospective study in Colombia

**DOI:** 10.1093/ofid/ofad500.1356

**Published:** 2023-11-27

**Authors:** Yeimer Ortiz-Martínez, Leidy Herrera-Caviedes, Claudia Figueroa-pineda, María J Melo-Amaya, Andrés F Henao Martínez

**Affiliations:** Universidad Industrial de Santander, Bucaramanga, Santander, Colombia; Universidad Industrial de Santander, Bucaramanga, Santander, Colombia; Universidad Industrial de Santander, Bucaramanga, Santander, Colombia; Universidad Industrial de Santander, Bucaramanga, Santander, Colombia; University of Colorado Anschutz Medical Campus, Aurora, CO

## Abstract

**Background:**

Vitamin D deficiency is prevalent in people living with HIV (PLWH) and is linked to various negative health outcomes. Despite numerous studies in other regions, information regarding vitamin D status and its determinants among PLWH in Latin America is scarce. This study aimed to determine the frequency of vitamin D deficiency in hospitalized patients with HIV in Colombia and identify associated risk factors.

**Methods:**

We conducted a prospective cohort study of hospitalized patients with HIV at a tertiary university hospital in Bucaramanga, Colombia, between December 2022 and April 2023. Serum 25-hydroxyvitamin D (25(OH)D) levels were measured at admission and categorized as suboptimal (≤30 ng/ml), insufficient (≤25 ng/ml), deficient (≤20 ng/ml), or severely deficient (≤10 ng/ml). Demographic, clinical, and paraclinical data were collected, and multivariate logistic regression was performed to identify factors associated with vitamin D deficiency.

**Figure:**
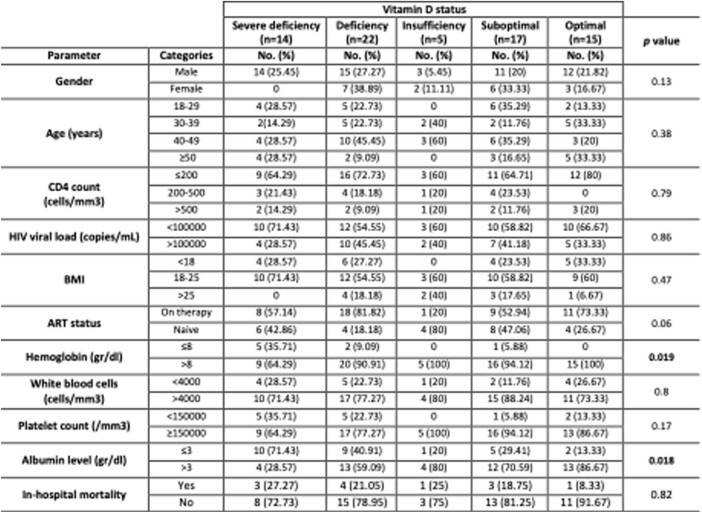

Table 1

**Results:**

A total of 73 PLWH were included in the study. 75.3% were men with a mean age of 40.6 ± 11.85 years. 50.6% were on antiretroviral therapy (ART) at the time of admission. 69.8% had a CD4 count ≤ 200 cells/mm3, and 63% had a viral load > 10,000 copies/ml. The in-hospital mortality was 19.35%. The median (IQR) 25(OH)D level was 21.28 ng/mL (13-27.8). Vitamin D deficiency was present in 49.3% of the participants, with 19.1% having severe deficiency. Insufficiency and suboptimal levels were found in 56.1% and 79.4%, respectively. No significant association was found between vitamin D deficiency and mortality or HIV-related factors, including ART, viral load, and CD4 count (Table 1). However, after multivariate analysis, albumin less than 3 g/dL was significantly associated with vitamin D deficiency (aOR 3.86, 95% CI: 1.37-10.85; *p*=0.01).

**Conclusion:**

This is the first prospective study in Colombia that highlights the high frequency of vitamin D deficiency in hospitalized patients with HIV. The finding of a significant association between hypoalbuminemia and vitamin D deficiency suggests that malnutrition or TNF-mediated cachexia may be contributors to this problem. Further studies are needed to explore the implications of vitamin D deficiency in this population and to develop appropriate interventions.

**Disclosures:**

**All Authors**: No reported disclosures

